# Factors associated with neonatal near-miss in selected hospitals of Gamo and Gofa zones, southern Ethiopia: nested case-control study

**DOI:** 10.1186/s12884-019-2684-x

**Published:** 2019-12-21

**Authors:** Abera Mersha, Agegnehu Bante, Shitaye Shibiru

**Affiliations:** Department of Nursing, College of Medicine and Health Sciences, Arba Minch University, Arba Minch, Ethiopia

**Keywords:** Neonatal near-miss, Nested case-control study, Selected hospitals, Gamo and Gofa zones, Southern Ethiopia

## Abstract

**Background:**

To scale up a comprehensive way of implementation to reduce neonatal mortality evaluation of factors for neonatal near-miss cases is very important. Certain studies were done in assessing near-miss cases, but they failed in identifying the proximate factors affecting profoundly. So, this study is to fill those gaps in the aforementioned studies, in assessing the factors affecting neonatal near-miss cases.

**Methods:**

A nested case-control study was conducted in selected three Hospitals of Gamo and Gofa Zones, Southern Ethiopia from April 5, 2018, to March 5, 2019. The structured standard tool was used to identify neonatal near-miss cases. Data were entered into Epi data version 3.1 and exported to Stata version 15 for analysis. A conditional logistic regression model was used to identify factors associated with near-miss cases. The goodness of fit was tested by a log-likelihood ratio (LR). In this study *P*-value < 0.05 was considered to declare a result as a statistically significant association.

**Results:**

In this study 121 neonatal near-miss cases, and 363 controls were involved. The identified factors that affect neonatal near-miss were multiparty (AOR = 3.81, 95%CI: 1.72, 8.42), antenatal care follow up (AOR = 0.02, 95%CI: 0.01, 0.05), premature rupture of membrane (AOR = 3.40, 95%CI: 1.53, 7.55), non-vertex presentation (AOR = 2.83, 95%CI: 1.44, 5.58), and cesarean delivery (AOR = 4.89, 95%CI: 2.34, 10.24).

**Conclusions:**

Those identified factors are better should be intervened. Strengthening antenatal care services by providing appropriate information for the mother and counseling about the consequences of multiparty and providing information on family planning. There is a need to identify, screen and critical follow high-risk mothers and give immediate and appropriate intervention as early as possible.

## Background

Globally, the neonatal mortality rate was decreased to 18 per 1000 live births and as indicated in many studies the main causes of neonatal deaths were preterm birth, intrapartum related complications, and sepsis [1–5]. In spite of developed nations, neonatal morbidity, and mortality rates remained high in resource-limited countries. It is projected that the number of survivors from a “neonatal near-miss” event is three to six times higher than the number of neonatal deaths [6].

Neonatal mortality is the utmost in resource-limited countries [1, 2]. Each year, at least 1.16 million African babies die in the neonatal period. Thirty-eight percent (38%) of babies in sub-Saharan Africa die of infections, mainly after seven days postpartum [7]. In Ethiopia, greater than 3/5th of infants and 2/5th of under-five deaths were neonatal deaths [8]. Based on, 2019Ethiopia Mini Demographic Health Survey report, 30 babies die per thousand live births [9].

A substantial proportion of this neonatal mortality could be banned by the appropriate management of the neonate presenting life-threatening complications (neonatal near-miss) [10]. Assessing neonatal near-miss (NNM) cases give an all-inclusive assessment of risk factors, instant outcomes and predictive factors in neonates delivered from mother with different obstetric complications [11]. The use of the near-miss approach in neonatal health is a new idea in the direction of a pioneering tool to improve the quality of perinatal care [12]. The NNM approach provided information that could be valuable to sightsee the quality of care issues and set priorities for in-depth assessments and health care improvements in newborn health [10, 12, 13]. Discussions of morbidity are better accepted than death case reviews by health care teams. Thus, the quality of care would improve. There is an expectation of the development of prospective surveillance processes of severe neonatal morbidity [14].

Evidence showed that the NNM rate was higher than the neonatal mortality rate; increasing 2.6 to 8 fold [10, 12, 15, 16]. The incidence of neonatal near-miss cases was ranged from 21.4 to 85.5 per 1000 live births [10–12, 15–19]. Different studies suggested the majority of neonatal near-miss cases were caused by respiratory dysfunction/failure, immunologic dysfunction/ failure, infections, central nervous system dysfunction/failure, birth asphyxia and very low birth weight [11, 20]. Compared to the neonatal mortality rate, more cases of NNM were observed among obstetric cases in which asphyxia, trauma or antepartum hemorrhage had occurred [11, 15].

Based on previous shreds of evidence Global Maternal Child Survival Program gives attention to newborns in Ethiopia. The package ropes the government of Ethiopia to improve community-based maternal neonatal and child health (MNCH) services to upsurge health-seeking behaviors; increases the delivery of eminence newborn care practices including management of newborn sepsis; and strengthens the supportive systems with a focus on district capacity building [21]. This program is underway, but to scale up a comprehensive way of implementation to reduce neonatal mortality evaluating determinants for NNM cases is very important. Some studies were done in assessing NNM cases, but they failed in identifying the proximate factors affecting intensely. So, this study is to fill those gaps of the aforementioned studies, in assessing the factors affecting NNM cases in selected Hospitals of Gamo and Gofa Zones, Southern Ethiopia.

## Methods

### Study setting and period

This study was conducted in selected three Hospitals of Gamo and Gofa Zones, Southern Ethiopia from April 5, 2018, to March 5, 2019. Gamo and Gofa are Zones within the South Nations, Nationalities and Peoples’ Region (SNNPR) of Ethiopia. There are seven hospitals in Gamo and Gofa Zones but the study was done in selected three hospitals (Arba Minch General Hospital (AMGH), Sawla General Hospital (SGH) and Chencha Primary Hospital (CPH)). The total population of the study area is 2,019,687. The estimated number of women of reproductive age (15–49) is 470,587 from this, the estimated number of delivery is 69,881 and the estimated number of live birth is 69,881. In Gamo and Gofa Zones, the skilled delivery rate is 51.2% [22, 23].

### Study design

A nested case-control study matched for sex and study setting (hospitals) were employed to meet the objectives.

### Selection of cases and controls

The neonatal near-miss cases were identified by using standard definition, and appropriate criteria and defined as the presence of at least one pragmatic marker or management severity criteria [14, 19]. The cases were matched for sex and study setting (hospitals) with controls that for each case three controls with similar sex groups and study settings were selected.

### Exclusion criteria

Multiple pregnancies, neonatal deaths, and neonates who were referred from other health care institutions that were out of the study hospitals were excluded.

### Sample size determination

The sample size was computed by using sample size determination in an unmatched case-control study in the Epi info7 software Stat Cal. The following assumptions were considered: 95% level of confidence, power of 80%, the ratio of cases to control 1:3 and percent of controls exposed 15.8 and percent of cases with exposure 5.4. The percent of cases and controls were taken from the study conducted in northeastern Brazil that the most determinate variables for the neonatal near-miss were maternal age greater than 35 or advanced age [24]. Base on the above assumptions the estimated sample size was 110 cases and 330 controls. After considering the non-response of 10%, the final sample size used for this study was 121 cases and 363 controls.

### Data collection method

A pre-tested, structured interviewer-administered questionnaire and standard abstraction checklist to review data from medical records were used to collect the data. The standardized data abstraction form developed by the London School of Hygiene and Tropical Medicine (LSHTM) and its partners in multi-country near-miss projects in Francophone Africa, the World Health Organization (WHO) Multi-Country Survey Project on severe maternal morbidity and the Unmet Obstetric Need (UON) projects led by the Institute of Tropical Medicine in Antwerp [25] were used to abstract pertinent information (Additional file 1). Neonates who experienced neonatal near-miss cases were identified by well-trained six diploma holders midwives and supervised by two MSc holder nurses. The data were collected from the delivery ward, postnatal ward and neonatal intensive care unit (NICU) of each hospital.

### Criteria’s to identify near-miss cases

The description of the criteria to identify neonatal near-miss cases were stated in detail below (Table 1).

**Table 1:** Criteria to identify neonatal near-miss cases in selected Hospitals of Gamo and Gofa Zones, Southern Ethiopia, 2018/9

Criteria	Descriptions
Pragmatic markers criteria	It is the severity of a criterion that is used to classify neonate as a neonatal near-miss. It includes birth weight < 1750 g, Apgar score < 7 at 5 min and GA < 33 weeks [14].
Management severity criteria	It is a criterion based on the management base. It includes parenteral antibiotic therapy, nasal continuous positive airway pressure (CPAP), any intubation, and phototherapy within 24 h. of life, cardiopulmonary resuscitation (CPR), use of vasoactive drugs, anticonvulsants, surfactant, blood products, steroids for the treatment of refractory hypoglycemia, surgery, use of antenatal steroid, use of parenteral nutrition, identification of congenital malformation according to the ICD-10 if considered a near-miss case by another criterion and admission to the NICU [14].

### Data quality control

To ensure the quality of the data, the questionnaires in the local language were used to collect the information from the study participants. All health care workers working in the delivery ward, postnatal ward, and neonatal intensive care unit in each participating hospital were also sensitized to the issue so that they were informed the enumerators when they suspected near-miss cases. Besides, the inclusion criteria for the neonatal near-miss was printed and posted on the wall of each ward at all participating hospital. The data collectors were daily visits to the delivery ward, postnatal ward and NICU to check for potential cases. The data collectors were trained to standardize methods and ensure the consistency of data collection. Handy and frequent supervision of the questionnaire was made to ensure completeness and consistency.

### Data analysis and processing

Data were coded and entered into Epi data version 3.1 and then exported to Stata version 15 for analysis. The data were cleaned before analysis. Bivariate analysis was done by using a binary logistic regression model to see the association between each independent variable and neonatal near-miss cases. The goodness of fit was tested by a log-likelihood ratio (LR). To include the variables into the final model *P* < 0.25 in the bivariate analysis, and context point of view were considered. A conditional logistic regression model was used to ascertain the independent effect. Multi co-linearity was checked by collinearity statistics (Variance inflation factor and Tolerance). A crude and adjusted odds ratio with 95%CI was estimated to identify factors affecting NNM cases. In this study *P*-value < 0.05 was considered to declare a result as a statistically significant association.

## Results

### Socio-demographic and economic characteristics

In these 121 cases and 363 controls were involved with a response rate of 100% for both cases, and controls, as it was a nested case-control study. The mean age in years and a standard deviation of neonate’s mother was 29.95 ± 4.12 for cases and 27.61 ± 6.65 for controls. The majority (96.7%) of the neonate’s mother for cases and 361 (99.4%) for controls were married. Of the respondents, 104 (86.0%) for cases and 301 (82.9%) for controls were Gamo ethnicity. Regarding the educational status of neonates mother 66 (54.5%) for cases and 207 (57.0%) for controls had secondary education (grade 9 to 12). Fifty (41.3%) of neonates’ fathers had the educational status of secondary for cases and 162 (44.6%) for controls. Out of neonates mother 71(58.7%), and 45(37.2%) had orthodox, and protestant religion followers for cases and 172(47.4%) and 180(49.6%) for controls respectively. Twenty-nine (24.0%) of the neonates’ mother was a housewife for cases and 214 (59.0%) for controls. Of the respondents, 79(65.3%) for cases and 266(73.3%) for controls were urban residents (Table 2).

**Table 2:** Socio-demographic and economic characteristics of study participants in selected hospitals of Gamo and Gofa Zones, Southern Ethiopia, 2018/9

Characteristics	Cases(*n* = 121)	Control(*n* = 363)	COR(95%CI)	*P*-value
Age				
15–24	6 (5.0)	114 (31.4)	1	
25–34	92 (76.0)	188 (51.8)	9.29 (3.94,21.93)	< 0.0001
≥ 35	23 (19.0)	61 (16.8)	7.16 (2.77,18.54)	< 0.0001
Marital status				
Married	117 (96.7)	361 (99.4)	0.16 (0.03,0.89)	0.04
Other^a^	4 (3.3)	2 (0.6)	1	
Ethnicity				
Gamo	104 (86.0)	301 (82.9)	1.56 (0.70,3.45)	0.28
Gofa	9 (7.4)	26 (7.2)	1.56 (0.53,4.58)	0.42
Other**†**	8 (6.6)	36 (9.9)	1	
Educational status of the mother				
No formal education	12 (9.9)	32 (8.8)	1	
Primary (1–8)	21 (17.4)	56 (15.4)	1.00 (0.44,2.29)	1.00
Secondary (9–12)	66 (54.5)	207 (57.0)	0.85 (0.41,1.75)	0.66
College and above	22 (18.2)	68 (18.7)	0.86 (0.38,1.96)	0.72
Occupation of the mother				
Housewife	29 (24.0)	214 (59.0)	1.69 (0.38,7.53)	0.49
Merchant	75 (62.0)	42 (11.6)	22.32 (5.04,98.94)	< 0.0001
Government employer	15 (12.4)	82 (22.6)	2.29 (0.49,10.69)	0.29
Other®	2 (1.7)	25 (6.9)	1	
Educational status of the father				
No formal education	16 (13.2)	31 (8.5)	1	
Primary (1–8)	17 (14.0)	69 (19.0)	0.48 (0.21,1.07)	0.07
Secondary (9–12)	50 (41.3)	162 (44.6)	0.59 (0.30,1.18)	0.14
College and above	38 (31.4)	101 (27.8)	0.73 (0.36,1.48)	0.38
Occupation of the father				
Farmer	38 (31.4)	118 (32.5)	0.63 (0.35,1.16)	0.14
Merchant	16 (13.2)	66 (18.2)	0.48 (2.23,0.98)	0.05
Government employer	42 (34.7)	130 (35.8)	0.63 (0.35,1.15)	0.13
Other©	25 (20.7)	49 (13.5)	1	
Place residence				
Urban	79 (65.3)	266 (73.3)	0.69 (0.44,1.07)	0.09
Rural	42 (34.7)	97 (26.7)	1	
The average income per month				
< 35.4USD	38 (31.4)	39 (10.7)	1	
35.4–88.5USD	67 (55.4)	232 (63.9)	0.29 (0.18,0.50)	< 0.0001
> 88.5USD	16 (13.2)	92 (25.3)	1.18 (0.09,0.36)	< 0.0001

^a^single, divorced and separated due to work, **†**Zayise, Amhara, Oromo, Gurage, Woliata, Konso, Derashe, Oyida and Gidicho,®daily laborer and student, and©waiver and daily laborer

### Maternal and child health, and obstetric factors

Of the neonates’ mother, 101(83.5%) for cases and 196(54.0%) for controls had multipara (birth order ≥2). Five (5.0%) of the respondents had a history of stillbirth, and 5(5.0%) had a history of abortion for cases, and 38(19.4%) and 39(19.9%) for controls respectively. Regarding birth interval, 37(36.6%) of the mothers of the neonates were birth inter of 24–48 months for cases and 136 (69.4%) for controls. Thirty-two (31.7%) of the neonates’ mother had a history of neonatal death for cases and 49(25.0%) for controls. Of the neonates’ mothers, 54 (44.6%) had antenatal care (ANC) follow up and 121(100%) had postnatal care for cases and 337(92.8%) and 363(100%) for controls respectively. Regarding the mode of delivery, 57(47.1%) gave birth by cesarean, and 64(52.9%) were by non-cesarean for cases, and 49(13.5%), and 314(86.5%) for controls respectively. Sixty-three (52.1%) of the neonates’ presentation during delivery was vertex for cases and 325(89.5%) for controls (Table 3).

**Table 3:** Maternal and child health and obstetric factors of study participants in selected hospitals of Gamo and Gofa Zones, Southern Ethiopia, 2018/9

Variables	Cases (*n* = 121)	Controls (*n* = 363)	COR(95%CI)	*P*-value
Party				
Primipara	20 (16.5)	167 (46.0)	1	
Multipara	101 (83.5)	196 (54.0)	4.30 (2.55,7.25)	< 0.0001
ANC				
Yes	54 (44.6)	337 (92.8)	0.06 (0.04,0.11)	< 0.0001
No	67 (55.4)	26 (7.2)	1	
Number of ANC visit				
No visit	67 (55.4)	26 (7.2)	1	
1–3	34 (28.1)	124 (34.1)	0.11 (0.06,0.19)	< 0.0001
≥ 4	20 (16.5)	213 (58.7)	0.04 (0.02,0.07)	< 0.0001
Hemorrhage				
Yes	8 (6.6)	6 (1.7)	4.21 (1.43,12.39)	0.009
No	113 (93.4)	357 (98.3)	1	
Cause of Hemorrhage				
NA^a^	113 (93.4)	357 (98.3)	0.24 (0.05,1.08)	0.06
Placenta praevia	4 (3.3)	3 (0.8)	1.00 (0.12,8.31)	1.00
Other®	4 (3.3)	3 (0.8)	1	
Premature rupture of membrane				
Yes	63 (52.1)	41 (11.3)	8.53 (5.27,13.82)	< 0.0001
No	58 (47.9)	322 (88.7)	1	
Hypertension during pregnancy				
Yes	24 (19.8)	25 (6.9)	3.35 (1.83,6.12)	< 0.0001
No	97 (80.2)	338 (93.1)	1	
Classification of HTN				
NA^a^	97 (80.2)	338 (93.1)	0.22 (0.05,0.98)	0.05
Pre-eclampsia eclampsia syndrome	20 (16.5)	22 (6.1)	0.68 (0.14,3.43)	0.64
Chronic hypertension	4 (3.3))	3 (0.8))	1)	
Mode of delivery				
Cesarean	57 (47.1)	49 (13.5)	5.71 (3.58,9.10)	< 0.0001
Non-cesarean**±**	64 (52.9)	314 (86.5)	1	
Presentation				
Vertex	63 (52.2)	325 (89.5)	1	
Non-vertex**©**	58 (47.9)	38 (10.5)	7.87 (4.82,12.85)	< 0.0001

^a^Not applicable,**®**postpartum hemorrhage, accreta/increta/percreta, hemorrhage during delivery, uterine rupture and other obstetric hemorrhages, **±** spontaneous vaginal delivery, and instrumental (assisted) delivery, and**©**breech, transverse, face and brow

### Factors associated with neonatal near-miss

In the multivariable analysis of independent variables within conditional logistic regression model multiparty, ANC, premature rupture of membrane (PROM), non-vertex presentation and cesarean mode of delivery were significantly associated with NNM cases.

The odds of NNM were 3.81 among multiparous as compared to nulliparous (AOR = 3.81, 95%CI: 1.72, 8.42). A neonate’s mother who had ANC follow up was 98% times less likely to have NNM cases as compared to mothers hadn’t (AOR = 0.02, 95%CI: 0.01, 0.05). A neonate’s mother who faced PROM before the onset of delivery was 3.40 times, and who gave birth by cesarean mode of delivery were 4.89 times more likely to have NNM cases (AOR = 3.40, 95%CI:1.53, 7.55), and (AOR = 4.89, 95%CI: 2.34, 10.24) respectively. Neonates who had non-vertex presentation were 83% more likely to become near-miss as compared to counterparts (AOR = 2.83, 95%CI: 1.44, 5.58) (Table 4).

**Table 4:** Multivariable analysis of the factors associated with neonatal near-miss cases in the conditional logistic regression model in selected hospitals of Gamo Gofa Zones, Southern Ethiopia, 2018/9

Variables	Adjusted OR(95%CI)	*P*-value
Educational status of the mother		
Primary (1–8)	1.89 (0.53,6.76)	0.33
Secondary (9–12)	1.32 (0.43,4.08)	0.63
College and above	1.19 (0.34,4.23)	0.79
Educational status of the father		
Primary (1–8)	0.42 (0.13,1.44)	0.17
Secondary (9–12)	0.96 (0.33,2.78)	0.95
College and above	0.79 (0.26,2.41)	0.69
Place residence		
Urban	0.73 (0.35,1.54)	0.41
Party		
Multipara	3.81 (1.72,8.42)*	**0.001**
Antenatal care		
Yes	0.02 (0.01,0.05)*	**< 0.0001**
Hemorrhage		
Yes	0.56 (0.10,3.09)	0.51
Premature rupture of membrane		
Yes	3.40 (1.53,7.55)*	**0.003**
Hypertensive disorder during pregnancy		
Yes	1.08 (0.34,3.45)	0.89
Presentation		
Non-vertex**©**	2.83 (1.44,5.58)*	**0.003**
Mode of delivery		
Cesarean	4.89 (2.34,10.24)*	**< 0.0001**

**©**breech, transverse, face and brow presentation, and *significant at *P* < 0.05.

## Discussion

Identifying factors affecting neonatal near-miss cases is fundamental to condense neonatal mortality. Some studies were conducted in assessing neonatal near-miss cases, but they failed in identifying the most determinants for near-miss cases. Therefore, this study showed the most proximate factors affecting near-miss cases in the study settings.

Of the characteristics that were assessed in this study; multiparty, antenatal care, premature rupture of membrane, non-vertex presentation, and cesarean mode of delivery had a significant association with neonatal near-miss cases.

As indicated in this finding multiparty was significantly associated with NNM. This was in line with a study done in Ethiopia [26], but inconsistent with a study done in Southeast Brazil [19]. This difference is maybe due to differences in socioeconomic status and health care delivery system. Not having ANC follow-up had a significant factor for having neonates with a life-threatening condition (near-miss) as point outed in this study. This was congruent with a study done in Southeast Brazil [19]. This is obvious that the pregnant mother avoids preventable risk factors after having ANC follow and early identification, and treatment of pre-existing conditions, and early screening of conditions that occurred during pregnancy.

Premature rupture of membrane and cesarean mode of delivery were significantly associated with NNM cases in this finding. This was in line with studies done in South Africa, and three studies in Brazil [15, 16, 19, 27]. The reason for this is that those stated conditions are one, or in another way can affect the neonates during intra-uterine as well as extra-uterine life and predispose for life-threatening conditions. In this study, the non-vertex presentation had a significant association with NNM cases. This is the fact that mal-presentation during delivery cause different complications in the mother as well as in the baby, and results for neonatal near-misses.

In this study, hemorrhage and hypertensive disorder during pregnancy were not significantly associated with neonatal near-miss cases. This was incongruent with a study done by Kale et al. [19]. The reason for this discrepancy may be methodological aspects of the study (difference in the study participant selection, study setting, and design). Educational status of the mother and father and place of the residence had also not significate association with neonatal near-miss cases as speculated in this finding. This is because, nowadays there is an improvement in the health care delivery system, advances in technology, and seeking health information irrespective of residence and educational status.

The utmost importance of this study for public health is: identifying the potential factors that predispose the newborn for life-threatening (near-miss) conditions are very important to tackle the underlining causes and to give immediate solutions. The finding of this study initiates different stakeholders in the health care system to design appropriate strategies and planning for the measurements to be taken to avoid those potential factors, both in the health care institutions as well as in the community at large. This study becomes one input for health policymakers and program developers typical regarding neonatal health in the health care delivery system.

The limitation of this study is that it does not incorporate some of the variables that are addressed in the community, such as wealth index, nutritional status, and cultural aspects. Therefore, other scholars should consider those situations and it also very important if they supplement or triangulate with a qualitative study to dig out untouched aspects.

The strength of this study was that data on exposure and confounding collected before the occurrence of cases (neonatal near-miss case) (from the follow-up study conducted in the same setting), which reduces the potential for recall bias and uncertainty regarding the temporal sequence between exposure and case onset. Besides, it was more efficient to measure the exposure status as compared to the whole cohort.

In summary, there is a gap in previous studies to show the most proximate factors that affect neonatal near-miss cases. Therefore, this study envisioned to fill the aforementioned gaps. In this study, party, antenatal care, premature rupture of membrane, presentation, and mode of delivery were significantly associated with near-miss cases. Whereas, the educational status of the mother and father, place of residence, hemorrhage, and hypertensive disorder during pregnancy had not significate association with near-miss cases. The readers should consider the limitations of this study while interpreting the finding, and the other scholars will do more to overcome those limitations. The finding of this study gives an overriding reputation to tackle factors affecting neonatal health, which leads to near-miss and predisposing factors for neonatal mortality.

## Conclusions

Multiparty, not having antenatal care follow up, premature rupture of membrane, non-vertex presentation, and cesarean mode of delivery were the independently associated factors with neonatal near-miss cases. Strengthening antenatal care services by providing appropriate information for the mother, and counseling about the consequences of multiparty and providing information on family planning for those mothers. There is a need to identify, screen and critical follow high-risk mothers: those who had different complications during pregnancy, those who faced premature rupture of membrane, those who identified as the fetal presentation was non-vertex, and those undergoing cesarean section due to different indications.

## Supplementary information

**Additional file 1.** English version questionnaire (pdf)

## Data Availability

The data will not be shared to preserve participant anonymity.

## Abbreviations

ANC: Antenatal care
NICU: Neonatal intensive care unit
NNM: Neonatal near-miss
PROM: Premature rupture of membrane
