# Supplementary material for: Factors associated with neonatal near-miss in selected hospitals of Gamo and Gofa zones, southern Ethiopia: nested case-control study
Source: BMC Pregnancy Childbirth. 2019 Dec 21;19:516. doi: 10.1186/s12884-019-2684-x (PMC6925862; doi:10.1186/s12884-019-2684-x)
Supplement: Supplementary file 1 — Additional file 1. English version questionnaire (pdf) [file 12884_2019_2684_MOESM1_ESM.pdf]

English Version Questionnaire

Interview and Record Review ***Identification Number*** [ ]

SNo	Questions	Response	Skip
**Part I: Identifications**			
101	Hospital Name	a. AMGH<br>b. Chencha Primary Hospital<br>c. Sawla General Hospital	
102	Hospital code	____/___/____	
103	Date form filled to start	____/___/____	
104	Woreda/District	__________________________	
105	Kebele	__________________________	
106	Village	__________________________	
107	Phone number (anyone who are immediate response)		
108	Health Center in the surround		
109	Health Post in the surround		
110	Health extension worker name and phone number		
111	Admission date	_____/____/____EC	
112	Maternity record number/Medical Reg. Number	__________________	
113	Admission mode	a. Self-referred<br>b. Referred from another facility	
114	Means of transport used	a. Ambulance<br>b. Public transport<br>c. Personal vehicle<br>d. Other, specify____________	
**Part II: Socio-demographic and economic characteristics**			
201	How old are you?	____________(in completed year)	
202	What is your marital status?	a. Married<br>b. Single<br>c. Widowed<br>d. Divorced<br>e. Separated due to work	
203	What is your ethnicity?	_______________________	
204	What is mother's educational status?	a. No formal education<br>b. Primary(1-8)<br>c. Secondary(9-12)<br>d. College and above	
205	What is paternal educational status?	a. No formal education<br>b. Primary (1-8)	

		c. Secondary (9-12)<br>d. College and above	
206	What is your religion?	a. Orthodox<br>b. Catholic<br>c. Protestant<br>d. Muslim<br>e. Traditional	
207	What is the occupation of the mother?	a. House wife<br>b. Merchant<br>c. Government employer<br>d. Daily laborer	
208	What is the occupation of the father?	a. Farmer<br>b. Merchant<br>c. Government employer<br>d. Wavier<br>e. Daily laborer	
209	Where is the place of residence?	a. Urban<br>b. Rural	
210	How much is your family average income per month?	______________(ETB)	
**Part III: Maternal Factors related questions (Record review if it is available, if not interview the mother)**			
301	Number of pregnancies (gravidity)?	___________(in number)**If primi** →	**401**
302	Number of birth orders (parity)?	___________ (in number)	
303	Do you pervious history of still birth?	a. Yes<br>b. No	
304	Do you pervious history of abortion?	a. Yes<br>b. No	
305	The duration between the current birth and the preceding birth in months?	___________ (in months)	
306	Do you have pervious history of neonatal death?	a. Yes<br>b. No	
**Part IV: Maternal and child health care questions (similar with part III)**			
401	Did the woman receive antenatal care?	a. Yes<br>b. No →	**403**
402	If yes, number of visits	________________	
403	Do you have immediate PNC visit?	a. Yes<br>b. No	
**Part V: Obstetric related questions (Record review/interview)**			
501	Date of delivery or end of pregnancy	____/___/____	
502	Mode of delivery	a. Spontaneous vaginal delivery<br>b. Instrumental assisted delivery<br>c. Caesarean section	

503	Hemorrhage	a. Yes<br>b. No →	**505**
504	If, yes	a. Placenta praevia<br>b. Placenta accreta/increta/percreta<br>c. Retro-placental haematoma<br>d. Other first trimester hemorrhage<br>e. Hemorrhage during delivery (no other specification)<br>f. Uterine rupture<br>g. Postpartum hemorrhage (no other specification)<br>h. Other obstetric hemorrhage	
505	Premature rupture of member	a. Yes<br>b. No	
506	Hypertension	a. Yes<br>b. No →	**508**
507	If, Yes	a. Pre-eclampsia<br>b. Eclampsia<br>c. HELLP<br>d. Chronic hypertension<br>e. Gestational hypertension	
508	Anaemia (Hb < 11g/dl)	a. Yes<br>b. No →	**510**
509	If, Yes		
	a. Haemoglobin level (g/dl)	____________	
	b. Unknown		
510	Infection	a. Yes<br>b. No →	**512**
511	If, Yes	a. Unspecified infection<br>b. Puerperal endometritis<br>c. Pyelonephritis<br>d. Septicaemia<br>e. Peritonitis<br>f. Parietal suppuration<br>g. Malaria<br>h. Syphilis<br>i. Other systemic infection	
512	Dystocia	a. Yes<br>b. No →	**514**
513	If, Yes	a. Uterine pre-rupture<br>b. Prolonged labour<br>c. Foeto-pelvic disproportion	
514	Other pathologies	a. Yes<br>b. No →	**516**

515	If, Yes	a. HIV/AIDS<br>b. Embolic diseases (thrombosis/amniotic fluid or gaseous embolism<br>c. Heart disease<br>d. Sickle-cell disease<br>e. Other (specify: ___________)	
516	**Newborn**		
	**A.** Presentation	a. Chephalic<br>b. Breech<br>c. Transverse/face/brow<br>d. Other, specify___________	
	**B.** Sex	a. Male<br>b. Female<br>c. Unknown	
	**C.** Birth trauma	a. Yes<br>b. No	
	**D.** If the above response is yes	Specify, ________________	
	**E.** Baby referred to another facility?	a. Yes<br>b. No	
	**F.** Admitted to special care or intensive care unit?	a. Yes<br>b. No	

Identification criteria for Neonatal near miss

Put "√" for the management criteria on each phase if it is ok in the box

**Part VI: Neonatal Near-Miss Criteria's**				
**Pragmatic Markers**				
601	Gestational age	______(in week)		
602	Birth weight	______(g)		
603	Apgar score at 5th min	_______		
**Management Severity Criteria's**				
604	Use of intravenous antibiotics			
605	Nasal CPAP			
606	Any intubation			
607	Use of phototherapy in the first 24 hour			
608	Cardio pulmonary resuscitation			
609	Use of any vasoactive drug			
610	Use of anticonvulsants			
611	Use of surfactant			
612	Transfusion of blood derivatives			
613	Use of corticosteroid for treatment of refractory hypoglycemia			
614	Any surgical procedure			
615	Use of antenatal steroid			
616	Parenteral nutrition			
617	Congenital malformation – ICD-10			
618	Admission to NICU			
**Is the newborn considered near-miss?**			**a. Yes**<br>**b. No**	

Thanks!
